# The ups and downs of caloric restriction and fasting: from molecular effects to clinical application

**DOI:** 10.15252/emmm.202114418

**Published:** 2021-11-15

**Authors:** Sebastian J Hofer, Didac Carmona‐Gutierrez, Melanie I Mueller, Frank Madeo

**Affiliations:** ^1^ Institute of Molecular Biosciences NAWI Graz University of Graz Graz Austria; ^2^ BioHealth Graz Graz Austria; ^3^ BioTechMed Graz Graz Austria

**Keywords:** caloric restriction, fasting, healthspan, intermittent fasting, lifespan, time‐restricted eating, Autophagy & Cell Death, Metabolism

## Abstract

Age‐associated diseases are rising to pandemic proportions, exposing the need for efficient and low‐cost methods to tackle these maladies at symptomatic, behavioral, metabolic, and physiological levels. While nutrition and health are closely intertwined, our limited understanding of how diet precisely influences disease often precludes the medical use of specific dietary interventions. Caloric restriction (CR) has approached clinical application as a powerful, yet simple, dietary modulation that extends both life‐ and healthspan in model organisms and ameliorates various diseases. However, due to psychological and social‐behavioral limitations, CR may be challenging to implement into real life. Thus, CR‐mimicking interventions have been developed, including intermittent fasting, time‐restricted eating, and macronutrient modulation. Nonetheless, possible side effects of CR and alternatives thereof must be carefully considered. We summarize key concepts and differences in these dietary interventions in humans, discuss their molecular effects, and shed light on advantages and disadvantages.

## Graphical abstract

Can dietary modulations promote better health? The current article comprehensively reviews key concepts of dietary interventions, their clinical application and the beneficial and disadvantageous effects of caloric restriction and fasting on human health.

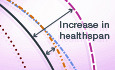

**Glossary**
Alternate day fasting (ADF): Alternate day fasting (ADF) is defined by an alternating sequence of fasting days (zero calorie intake) and eating days (ad libitum food consumption)
Alternate day modified fasting (ADMF): Like ADF, but allows <25% calorie intake on fasting days
AMP‐activated protein kinase (AMPK): A phylogenetically conserved nutrient‐ and energy‐sensing enzyme. AMPK becomes activated by events that increase the AMP/ATP ratio (e.g., exercise, fasting)
Caloric Restriction (CR): CR involves the reduction in calorie intake over a given period, while maintaining adequate levels of macro‐ and micronutrients
Caloric restriction mimetics (CRM): CRMs are pharmacologically active compounds that mimic some anti‐aging effects of CR
Circulating metabolome: The metabolome is a highly dynamic system and an important entity during CR and fasting. Moreover, it serves signaling functions, transports fuels, and contributes to the systematic health benefits during CR and IF
Fasting‐mimicking diet (FMD): The FMD is an eating regimen defined by a specific macro‐ and micronutrient composition that aims at inducing fasting‐like benefits
Healthspan: Healthspan is the period of life that individuals spend in good health without any aging‐related chronic diseases or disabilities
Intermittent Fasting (IF): IF refers to many different fasting regimes, which combine phases of restricted calorie intake and ad libitum consumption
Ketone Bodies (KBs): KBs (β‐hydroxybutyrate, acetoacetate, and acetone) are compounds produced by the liver upon fasting or caloric restriction. KBs serve as fuel and signaling molecules under low glucose levels
Lean body mass: The term “lean body mass” refers to non‐adipose tissue mass
Mechanistic Target of Rapamycin (mTOR): mTOR regulates fundamental processes like eukaryotic cell growth, protein synthesis, or autophagy as well as the metabolism by transducing environmental inputs, including nutrient availability. Deregulation of mTOR signaling is implicated in the progression of different diseases as well as in the aging process
Metabolic switch: The metabolic switch upon fasting is the point of negative energy balance, at which liver glycogen stores are depleted. It is characterized by lipid metabolization, free fatty acid usage, and production of KBs
Starvation: Starvation is a pathological state that results from a severe or total lack of nutrients
Time‐restricted eating (TRE): TRE describes the time‐limited consumption of calories during a period of 6–8 hours per day

## Introduction

According to the World Health Organization, life expectancy has been continuously increasing during the past decades (World Health Organization, 2019), although the pace of this increase seems to slow down globally (Cardona & Bishai, 2018). This development is accompanied by a rise in age‐associated diseases, including obesity, cardiovascular diseases (CVDs), type 2 diabetes, neurodegenerative diseases, and cancer. Thereby, our healthspan, defined as the life time without significant age‐related disease burden, increases at a slower pace than lifespan, resulting in more life years suffering from one or multiple diseases (World Health Organization, 2019). This is not only detrimental at a personal level but is also severely challenging health care systems.

### Dietary interventions for healthy aging

**Figure 1:**
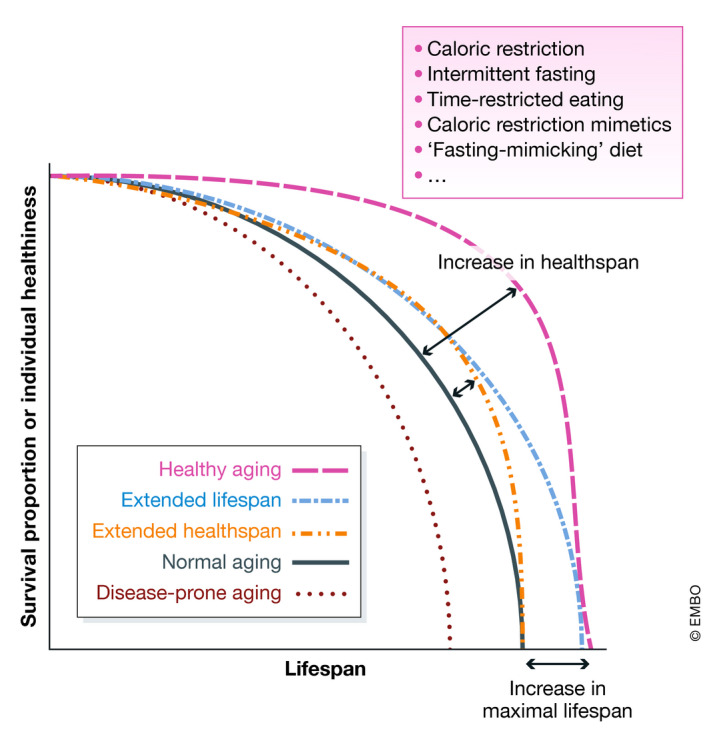
Theoretical concepts underlying dietary healthy aging intervention Different courses of aging, and how dietary approaches might influence the curves. Y‐axis shows survival proportions of a population and theoretical, individual healthiness. X‐axis shows the relative lifespan.

A major goal of aging research is to extend healthspan and delay the onset of age‐associated frailty and diseases. This concept has been termed “compression of morbidity” (Fries, 1980), or also “healthy aging,” and integrates various research fields to address the molecular and physiological modulation of aging, social well‐being, physical and cognitive fitness, etc. (Longo *et al*, 2015, 2021; Campisi *et al*, 2019; de Cabo & Mattson, 2019; Daniel, 2020; Eckstrom *et al*, 2020; Sharda *et al*, 2020). Theoretically, health‐ and life‐prolonging interventions can take different routes (Figure 1): (i) increasing maximal lifespan, while not affecting the ratio of healthy to unhealthy years; (ii) increasing healthspan only, without affecting maximal lifespan; and (iii) increasing both health‐ and maximal lifespan. On the other hand, (iv) increased disease burden affects health‐ and/or lifespan negatively (Figure 1). While genetic approaches in model organisms have revealed many molecular instances of aging control, their implementation in humans presents yet practical challenges and ethical limitations. However, recent advances in the field of gene editing via CRISPR‐Cas9‐technologies may soon become relevant, at least in some age‐associated diseases (Caobi *et al*, 2020). Non‐genetic health‐promoting interventions based on dietary modulation are less invasive and closer to broad‐scale application in health care. In fact, nutrition is a crucial determinant of aging. While chronic hypernutrition (as experienced in many western countries) leads to detrimental consequences, including increased incidence of overweight and obesity, malnutrition and undernutrition can evoke starvation effects, decreasing health and negatively impacting lifespan.

Dietary interventions in aging commonly target various outcomes related to aging as a whole. (i) In model organisms, extension of health‐ and lifespan of wild‐type or disease models is the most substantial outcome to be measured. In interventional clinical studies, this is yet an unreachable measure, but indirect measures of aging, such as epigenetic clocks, are emerging as methods to indirectly quantify potential anti‐aging interventions in humans (Horvath, 2013; Fitzgerald *et al*, 2021). (ii) Secondly, such interventions are preventive, i.e., they can delay the onset or progress of age‐related and generally non‐communicable diseases and avert obesity from hypernutrition. (iii) Finally, anti‐aging interventions can be revertive, i.e., ameliorate persisting symptoms of and tackle manifest age‐associated diseases and obesity.

### A case for caloric restriction and fasting

Among the existing dietary anti‐aging interventions, the sustained reduction in caloric intake without malnutrition (calorie restriction, CR) is probably the most robust and best studied form of geroprotective dietary intervention in model organisms (Fontana, 2009; Green *et al*, 2021). In fact, Clive McCay and colleagues noted already nearly nine decades ago that CR could prolong the lifespan of rats (McCay *et al*, 1935). In humans, reduced calorie intake without malnutrition has been repeatedly shown to elicit systemic health benefits (Most *et al*, 2017a), e.g., upon natural CR as observed on the Okinawan island (Willcox *et al*, 2006), the Comprehensive Assessment of the Long‐term Effects of Reducing Energy Intake (CALERIE) studies (Rickman *et al*, 2011), the CR with optimal nutrition (CRON) society, the Biosphere 2 experiment (Walford *et al*, 2002), and a long‐term cohort of alternate day fasting (ADF) followers (Stekovic *et al*, 2019). Besides CR, different forms of intermittent fasting (IF) have gained scientific and public interest for their broad health‐promoting properties (Michalsen & Li, 2013; Longo *et al*, 2021). Interestingly, virtually all extant religions have incorporated recurring fasting rituals, e.g., the Lent before Easter (Christianity) or the fasting month of Ramadan (Islam).

Besides the caloric element, macronutrient content and balance represent additional important denominators in health‐promoting dietary interventions (Simpson *et al*, 2015; Solon‐Biet *et al*, 2015). Thus, dietary constitution might be equally important as calorie content (Wilder *et al*, 2013; Simpson *et al*, 2015; Green & Lamming, 2019). Industrial ultra‐processing of food also promotes excessive calorie consumption and weight gain (Hall *et al*, 2019), emphasizing the importance of a diet’s underlying calorie sources.

### Scope of the review

In this non‐systematic review, we summarize molecular targets, physiological effects, and potential uses of CR and various forms of IF in clinical settings. Furthermore, we highlight potential caveats of these interventions and provide an outlook on novel, less invasive CR‐mimicking dietary interventions.

## Common types of CR and fasting

**Figure 2:**
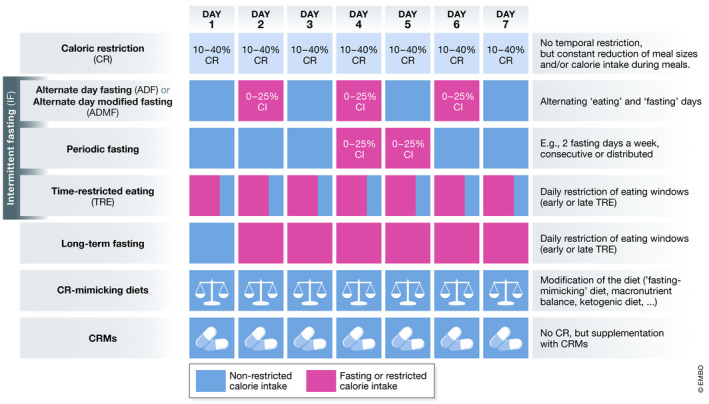
Different concepts of caloric restriction and fasting applications that have transitioned to clinical trials Blue = non‐restricted calorie intake, magenta = fasting or restricted calorie intake, CI = calorie intake, CRM = CR mimetic.

Besides CR, several health‐promoting dietary interventions have been developed, with often vaguely defined terminology, the most common of which are briefly described below (Figure 2).

- Caloric Restriction (CR) defines the reduction in calorie intake over a given period without malnutrition, sufficiently providing macro‐ and micronutrients. Typical levels of CR in mice and rats range from 10 to 50% (Mitchell *et al*, 2016; Acosta‐Rodríguez *et al*, 2017), while in humans most studies aim at 10–25% CR per day (Redman & Ravussin, 2011; Most *et al*, 2017b; Trepanowski *et al*, 2017). In rodent studies, CR is often achieved by restricting feeding windows and/or providing pre‐weighted amounts of food every day, which may be rapidly consumed by the animals (Acosta‐Rodríguez *et al*, 2017), thus possibly mixing effects of CR and IF. In humans, CR without significant temporal alterations of daily eating patterns can be achieved by reducing meal sizes. Of note, CR is often synonymously used with the term dietary restriction (DR), although DR rather describes the more specific restriction of macronutrients (e.g., proteins, carbohydrates, and amino acids) instead of overall food intake (Katewa & Kapahi, 2010).
- Intermittent Fasting (IF) is a loosely defined term that applies to different rhythmic, or recurring, arhythmic fasting regimens, in which calorie reduction is not achieved by reducing meal sizes, but rather by skipping one or multiple consecutive meals. Thus, all types of IF combine phases of restricted calorie intake and *ad libitum* consumption. However, if experimentally not controlled, craving‐induced overcompensation during the limited mealtimes may result in no or only minor calorie restriction.
  - Alternate Day Fasting, ADF, describes a form of IF, in which participants fast every other day (resulting in periodic fasting windows of ca. 36 h). Fasting days are followed by *ad libitum* eating days, most commonly with no restrictions. Fasting days may allow no calorie intake (Heilbronn *et al*, 2005), or—if performed more mildly—a baseline calorie intake of, e.g., 25% (Varady *et al*, 2009; Trepanowski *et al*, 2017; Gabel *et al*, 2019). In the latter case, the term alternate day modified fasting (ADMF) may be used (Anton *et al*, 2018). In non‐obese adults, ADF can result in CR of around 35%, as the calorie loss is not fully compensated on eating days (Stekovic *et al*, 2019). ADF without net energy reduction is possible (Templeman *et al*, 2021), but likely impracticable outside study settings.
  - Periodic Fasting (PF), often used synonymously with IF, describes a type of arrhythmic IF. The most prominent PF intervention is the so‐called “5:2” scheme (Scholtens *et al*, 2020), in which 2 days a week are calorie‐restricted to a minimal level, either sequentially or spread throughout the week. Other PF patterns might describe fasting phases of multiple consecutive days during a given timeframe (e.g., 5 consecutive fasting days per month).
  - Time‐Restricted Eating (TRE), in model organisms often termed time‐restricted feeding (TRF), describes the restriction of daily calorie intake to a consecutive time window of 6–8 h (Hatori *et al*, 2012; Gabel *et al*, 2018; Regmi & Heilbronn, 2020). TRE can be achieved by either skipping a specific meal (breakfast or dinner) or by compressing the meals into a narrow time window. In either case, TRE may be classified into early (e.g., skipping dinner) and late (e.g., skipping breakfast), depending on the first meal consumed. Compared to other IF protocols, TRE has a much higher frequency of fasting–eating cycles, thus shorter fasting windows, and may influence circadian rhythms more profoundly.
- Long‐term fasting, or very‐low‐calorie diet, is a loosely defined term often used for long‐lasting (more than 2 days, up to several weeks), very low calorie intake protocols (< 1,000 calories/day), which are often the basis for experimental, therapeutic, or lifestyle fasting applications (Wilhelmi de Toledo *et al*, 2020b).
- Other Fasting/CR‐mimicking dietary interventions comprise various experimental avenues that aim at eliciting (some) CR‐like molecular and physiological effects, such as the fasting‐mimicking diet (FMD; Brandhorst *et al*, 2015; Choi *et al*, 2016a; Wei *et al*, 2017), ketogenic diets (Ludwig, 2020), or low‐carbohydrate/fat diets (Kim, 2021).
- CR mimetics (CRMs) are pharmacologically active compounds that mimic some CR‐like effects on cells and organisms (Lane *et al*, 1998; Ingram & Roth, 2021). Examples include spermidine, rapamycin, metformin, or 2‐deoxy‐glucose (Mariño *et al*, 2014; Madeo *et al*, 2019; Ingram & Roth, 2021). Some of these candidates are under intense clinical investigation (Hofer *et al*, 2021). Depending on upstream or downstream molecular targets of CRMs, these compounds might provoke a broad or narrow selection of CR‐associated effects.

## Cellular and molecular events during CR and fasting

**Figure 3:**
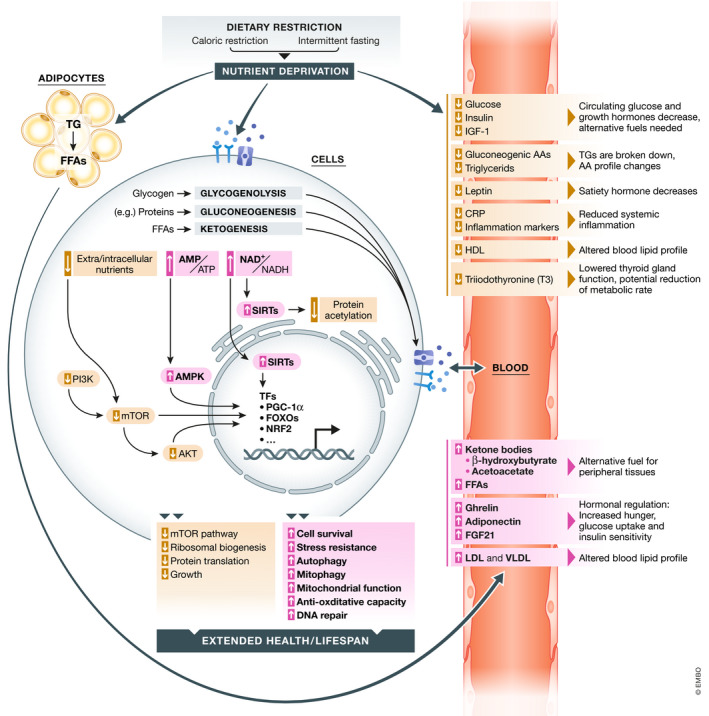
Key molecular events during CR and fasting at the cellular and metabolic levels CR and fasting act on multiple levels, eliciting an increase in cellular and organismal multi‐stress resistance, which leads to systemic health benefits. AKT = protein kinase B/PKB, AMPK = AMP‐activated protein kinase, AMP/ATP = ratio of adenosine monophosphate to adenosine triphosphate, BCAA = branched‐chain amino acid, FFA = free fatty acid, FGF21 = fibroblast growth factor 21, FOXO = forkhead box O, IGF‐1 = insulin‐like growth factor, (V)LDL = (very) low‐density lipoprotein, mTOR = mechanistic target of rapamycin, NAD^+^/NADH = ratio of oxidized to reduced nicotinamide adenine dinucleotide, NRF2 = nuclear factor erythroid 2‐related factor 2, PARP1 = Poly [ADP‐ribose] polymerase 1, PGC‐1α = peroxisome proliferation‐activated receptor gamma co‐activator 1α, TG = triglycerides.

The principal cellular mechanisms in response to nutrient availability are conserved from prokaryotes to primates and ensure survival under fasting conditions (Longo & Mattson, 2014). Here, we summarize key events and molecular players during fasting and CR in mammals, and which contribute to the systemic health‐promoting properties of these interventions (Figure 3).

### A metabolic switch ensures energy supply upon fasting

The fasting state is characterized by a metabolic switch from glucose dependency to lipid metabolization, free fatty acid (FFA) usage, and production of ketone bodies (KBs; b‐hydroxybutyrate, acetoacetate, and acetone) in the liver (although astrocytes might also produce KBs to fuel neurons; Guzmán & Blázquez, 2004). The occurrence and magnitude of this switch greatly depend on the specific intervention studied. Usually, it starts gradually after 12–36 h of food abstinence, depending on baseline hepatic glycogen storage, activity levels, and other factors. This event is marked by an increase in KBs that may continue up to several days until reaching a plateau (Anton *et al*, 2018; Steinhauser *et al*, 2018). During continuous CR with regular meals throughout the day, this switch is less likely turned on and/or dampened, depending on the duration and level of CR.

### Cellular processes and molecular targets affected by CR and fasting

Both during fasting episodes and CR, macro‐ and micronutrients are less available to cells and tissues. Thus, several pathways are similarly involved in mediating CR and IF effects. Most of the molecular events that govern cellular and molecular responses to nutrient deprivation have been studied in liver and muscle cells and may partially differ in other specialized cell types. All cells sense the availability of micro/micronutrients and react to either increased or decreased availability through various interconnected pathways, including those controlled by the serine/threonine protein kinase mechanistic target of rapamycin (mTOR) and AMP‐activated protein kinase (AMPK). mTOR is part of two complexes (mTORC1 and 2) that are both regulated by a wide variety of environmental signals and integrate those into cellular changes (Cornu *et al*, 2013; González & Hall, 2017; Liu & Sabatini, 2020). They are most prominently activated in response to amino acids and growth factors. AMPK, on the other hand, is activated upon low energy conditions and stimulates ketogenesis, fatty acid oxidation, glucose uptake, and inhibition of lipogenesis in various cell types (González *et al*, 2020). Accordingly, reduced glucose levels or diminished protein and amino acid availability—as induced via CR or fasting—lead to activation of AMPK and shutdown of mTOR, resulting in a reduction in protein synthesis and ribosomal biogenesis, as well as activation of autophagy. Likewise, low carbohydrate levels, on their part, are transduced via a decrease in insulin and IGF‐1 (insulin‐like growth factor 1), and reduced PI3K and AKT signaling. As a cellular consequence, mechanisms to cope with (nutritional) stress are upregulated via a regulatory network of transcription factors. These include processes such as DNA repair, autophagy, mitophagy, and oxidative stress defense, among others. From bottom up, the wide range of cellular alterations provoked by (periodic) nutrient depletion is believed to enhance cellular survival, reduce cellular senescence, improve organ function, counteract age‐associated deteriorations, decrease systemic problems, such as chronic inflammation, and consequently prolong organismal health‐ and lifespan.

Ultimately, CR‐ and fasting‐related cellular processes are regulated by a network of transcription factors. Among them are FOXO1 (forkhead box O1), which regulates gluconeogenesis, glycogenolysis and the cell cycle, as well as redox‐sensitive NRF2 (nuclear factor erythroid 2‐related factor 2), which activates a myriad of cytoprotective genes. In addition, sirtuins (SIRTs) are NAD^+^‐dependent histone deacetylases with multiple targets, also outside the nucleus, whose activity is tightly linked to the energy status (Haigis & Sinclair, 2010). For instance, in the liver, SIRT1 is stimulated during the metabolic switch and activates AMPK. SIRT1 also activates the transcription factors PPAR‐α (peroxisome proliferator‐activated receptor), which is important for hepatic lipid metabolism as well as ketogenesis, and PGC‐1α (peroxisome proliferation‐activated receptor gamma co‐activator), a master regulator of mitochondrial biogenesis. Active PGC‐1α coordinates enhanced mitochondrial function, while parallelly decreasing lipolysis and inhibiting gluconeogenesis, among many other effects. Intriguingly, during CR, SIRT1 has been found to be upregulated in various tissues of mammalian models and in the skeletal muscle of humans (Cantó & Auwerx, 2009; Rahman & Islam, 2011). Other sirtuin paralogs may also be relevant during the nutrient‐deprived states. For instance, SIRT3 regulates the activity of diverse mitochondrial enzymes and liver ketogenesis. Importantly, SIRT activity depends on NAD^+^, allowing SIRTs to sense the dietary status. In fact, an increased ratio of NAD^+^ to NADH (leading to SIRT activation) represents an important cellular event in the fasting state. Similarly, an increased ratio of AMP to ATP (also leading to AMPK activation) is also a characteristic event that mirrors the energy balance shift upon CR and fasting (Yang *et al*, 2007; Hasenour *et al*, 2013).

Another important cellular consequence of nutrient depletion is autophagy, a conserved intracellular recycling program that clears dysfunctional organelles and proteins. Its activity generally decreases during aging (Barbosa *et al*, 2019), and its dysregulation may contribute to neurodegenerative diseases, CVDs, and cancer (Schiattarella & Hill, 2016; Abdellatif *et al*, 2018; Yun & Lee, 2018; Barbosa *et al*, 2019; Corti *et al*, 2020; Park *et al*, 2020). In turn, the induction of autophagy by pharmacological (e.g., CRMs), genetic, or dietary (e.g., fasting and CR) means has been causally linked to health‐ and lifespan extension (Jia & Levine, 2010; Hansen *et al*, 2018; Madeo *et al*, 2019). Autophagy is activated and regulated via a complex regulatory network that involves the AMPK‐mediated control of mTOR, SIRTs, and protein acetylation. Deacetylation often favors autophagic processes and, in mice and humans, acute fasting has been shown to reduce acetylation of cytoplasmic and nuclear proteins, which depends on the availability of acetyl‐CoA (Pietrocola *et al*, 2017). Thus, many cellular and metabolic effects of fasting and CR converge on autophagy induction (Madeo *et al*, 2019).

### Changes in the circulating metabolome during acute fasting and CR, and maintenance of energy supply

The circulating metabolome during CR and fasting phases crucially contributes to the systemic health benefits, serves signaling functions, and transports fuel. The metabolome is a highly dynamic system that gradually changes upon duration and level of nutrient deprivation. Steinhauser *et al* (2018) identified distinct classes of metabolites that behave differently over a fasting period of 10 days in humans. Metabolic changes during CR will share many of these fasting‐associated changes, depending on length and magnitude of calorie deprivation.

One of the most prominent energetic reactions to fasting and drastic CR is lipid utilization. As outlined above, this results in increased blood stream levels of KBs and FFAs as alternative fuel types when glucose levels become low. Prior to lipid utilization, glucose is released from hepatic glycogen stores via glycogenolysis for usage in non‐hepatic tissue. Further glucose can be sourced from gluconeogenesis using glucogenic amino acids after protein breakdown, mainly in the liver and some other tissues. During the first days of fasting, branched‐chain amino acids are elevated in the blood stream. Subsequently, acetyl‐CoA from beta‐oxidation using adipocyte‐derived FFAs (released from triacyl‐ and diacylglycerols into the blood stream) build the basis for KB production in the liver. KBs are then released into the blood stream, reaching millimolar concentrations in the fasting state, and can be utilized by target organs/cells to obtain acetyl‐CoA via ketolysis to feed into the tricarboxylic acid cycle for ATP generation. This is especially important for energy‐demanding tissues like muscle and brain (Cahill, 2006). Notably, FFAs and KBs also harbor signaling functions on molecular targets like hormones and transcription factors with health‐promoting properties (Zechner *et al*, 2017). Interestingly, in model organisms, the sole supplementation of KBs can prolong health‐ and lifespan on its own (Camberos‐Luna *et al*, 2016; Veech *et al*, 2017).

Among other factors that usually show elevated blood stream levels upon fasting and CR is ghrelin (“hunger hormone”), which provokes the typical hunger sensation (Müller *et al*, 2015). This goes alongside with increased concentrations of the adipose tissue‐secreted hormone adiponectin, which increases glucose uptake, insulin sensitivity, and fatty acid oxidation. Another crucial player in the fasting response is the liver‐derived FGF21 (fibroblast growth factor 21), which stimulates glucose uptake in adipocytes among other effects (Geng *et al*, 2020).

On the contrary, several factors are depleted in the blood stream. These include insulin, IGF‐1, glucose, gluconeogenic amino acids, and triglycerides. As a counterplayer to ghrelin, leptin, a peptide hormone that inhibits hunger sensation, decreases. Furthermore, inflammatory markers and markers of oxidative stress decrease, including C‐reactive protein (CRP), a hepatic protein involved in the inflammatory acute‐phase response (Collet *et al*, 2017; Anton *et al*, 2018; Steinhauser *et al*, 2018). Importantly, these observations have been made in rodent as well as in human CR and fasting studies. This decrease in inflammatory markers may contribute to the systemic health‐promoting effects upon longer CR and IF studies, as seen in in model organisms and humans. Furthermore, cardiovascular blood markers change profoundly, including decreased triglycerides, elevated LDL species, and decreased HDL, contributing to the improvement of the cardiovascular system observed in CR and IF studies.

Several observational and interventional studies have shown reduced thyroid gland function in CR and IF cohorts. In agreement, a reduction in energy expenditure is often reported in CR studies, although these findings have been controversially discussed (Mitchell *et al*, 2017). In rodent studies, core body temperature (which is regulated by the thyroid gland) is often reported to decrease during CR and weight loss, and housing at thermoneutrality reverses some of CR’s (metabolic) effects (Guijas *et al*, 2020), arguing for some level of causal connection. In humans, plasma levels of the thyroid hormone triiodothyronine (T3) decrease in a non‐pathological range upon ADF (Stekovic *et al*, 2019) and CR (Soare *et al*, 2011; Fontana *et al*, 2016; Redman *et al*, 2018), a potential sign of slowed aging and metabolic rate (Rozing *et al*, 2010; Jansen *et al*, 2015). In line, energy expenditure and metabolic rate have been shown to decrease upon CR, even when adjusted for weight loss, although the outcome on body core temperature is mixed (Martin *et al*, 2007; Jiménez Jaime *et al*, 2015; Most *et al*, 2017a; Redman *et al*, 2018). Data from IF studies on these parameters are scarce and to date show no consistent changes (Lessan & Ali, 2019; Stekovic *et al*, 2019).

### CR and IF prolong the lifespan of model organisms

The assessment of how CR and IF may affect aging has to be framed within the complexity of the aging process itself. The nine “hallmarks of aging” were defined to reflect this complexity as a deteriorating process that affects organisms at all levels (López‐Otín *et al*, 2013). They include, for instance, mitochondrial dysfunction, loss of proteostasis, cellular senescence, and telomere shortening. Importantly, all of the latter have been shown to be counteracted by CR and IF in model organisms. In agreement with this, CR and IF interventions prolong health‐ and/or lifespan of yeast, worms, flies, and rodents. Non‐human primates also benefit from CR (30%), as shown in the University of Wisconsin study with rhesus monkeys, which reported improved survival and health (Colman *et al*, 2009, 2014). In contrast, a similar study conducted at the National Institute on Aging showed no lifespan benefits, although health parameters improved nearly significantly (Mattison *et al*, 2012). Beyond lifespan extension, it is the “compression of morbidity” which co‐defines the success of an anti‐aging intervention, and many age‐associated disorders that are ameliorated by CR in models have also been shown to be impacted in human studies (reviewed in (Green *et al*, 2021)). These include obesity, CVDs, cancer incidence, and type 2 diabetes, among others. Similar observations, although to a lesser extent, were made in human IF studies (Longo *et al*, 2021). All of the above discussed effects on cellular and organismal levels can contribute to the anti‐aging properties of CR and IF.

### Brief summary

The sum of metabolic, transcriptional, and proteomic alterations, but also those of the microbiota (briefly discussed later in the review) that are evoked by CR and fasting, result in increased stress resistance, enhanced cell and DNA repair, autophagy induction, and enhanced mitochondrial function. As a result, these alterations may impact several aspects of cellular and organismal health that lead to prolonged health‐ and lifespan in models and systemic health promotion in humans (Figure 3). However, the exact timescale of these effects and how they can be medically employed in a targeted manner are yet largely uncharted areas.

## Comparing CR and fasting interventions

To date, it is yet not fully understood if the respective effects observed upon mere CR and fasting can be seen as fully independent from each other. In fact, as many classic rodent CR studies achieve CR by reducing feeding time windows, effects in these studies may be elicited by both the CR and increased fasting phases. At the same time, there has been much debate whether intermittent energy restriction (IF) may harbor superior effects compared to continuous restriction (CR). For instance, rodents fed isocaloric, high‐fat, and high‐sucrose diets show delayed disease onset during aging and are largely protected from metabolic disturbances and weight gain when subjected to IF (Anson *et al*, 2003; Hatori *et al*, 2012; Sherman *et al*, 2012; Chaix *et al*, 2014, 2019; Kim *et al*, 2019; Mitchell *et al*, 2019). This suggests a partial uncoupling of IF‐induced health benefits from CR, highlighting the importance of periodic fasting episodes.

In humans, only few studies have addressed the challenge of isocaloric diets upon fasting or CR studies, as several intrinsic limitations impede such study designs. One study found no differences between calorie‐matched CR and ADMF (Trepanowski *et al*, 2017), similar to a further study that compared CR vs. zero‐calorie ADF (Catenacci *et al*, 2016). A small‐scale study with normal‐weighted participants compared isocaloric diets consumed in three meals or one meal and found a subtle reduction in body weight and fat mass as well as an increase in blood pressure and blood lipids in the 1 meal/day group (Stote *et al*, 2007). Most of the other blood parameters, as well as heart rate and body temperature, remained unchanged. A similar study found no differences in fasting levels of BDNF, insulin, leptin, ghrelin, and adiponectin, among others, but for the 1 meal/day group observed a delayed response in the oral glucose tolerance test, performed in the morning (Carlson *et al*, 2007). Moreover, a recent work compared 3 weeks of continuous CR (25%) with ADF (0 calories on fasting days and 150% on eating days, resulting in a theoretical net CR of 25%) and ADF without energy restriction (0 calories during fasting days and 200% calories on eating days; Templeman *et al*, 2021). Interestingly, weight loss was comparable between CR and 150%‐ADF, but the latter caused an unfavorable greater loss of lean mass. ADF without energy restriction, on the other hand, resulted in an attenuated weight loss (ca. −0.5 kg vs. −1.6 to −1.7 kg in the other groups; Templeman *et al*, 2021). Overall, ADF had no significant, superior benefits on metabolic parameters or body composition in this study. In line, 3 months of PF in overweight and obese adults do not provoke improved outcomes on circulating biomarkers, weight or fat loss when compared to a continuous, matched CR intervention (20%) (Schübel *et al*, 2018). On the other hand, PF for 4 months (2 fasting days/week during the first 3 months, 1 fasting day/week during the 4^th^ month), and allowing some calories on fasting days resulted in improved insulin sensitivity and greater fat loss in overweight women, when compared to continuous CR (Harvie *et al*, 2013). Whether weight loss rates differ between intermittent and continuous types of energy restriction is still under research, but growing evidence points to equal rates when duration and overall CR are matched (Harvie & Howell, 2017; Trepanowski *et al*, 2017). Meta‐analyses and systematic reviews support this notion (Varady, 2011; Cioffi *et al*, 2018). Altogether, the body of evidence available so far warrants further direct comparison studies between CR and IF.

The timing of eating windows, especially during TRE, may also influence the health outcome. Meta‐analyses have shown that breakfast skipping is associated with increased CVD risk and all‐cause mortality (Ofori‐Asenso *et al*, 2019; Chen *et al*, 2020). This suggests superiority of early eating windows during TRE, which in general has been shown to broaden health benefits (Longo & Panda, 2016). In line, a recent study of 3 months of late TRE (eating window 12 pm to 8 pm) found no significant improvements in weight loss, fasting insulin levels, fat mass glucose, or blood lipid levels (Lowe *et al*, 2020). Moreover, a 6‐week study of TRE without weight loss in non‐obese adults resulted in no significant change in cardiovascular parameters (Martens *et al*, 2020), suggesting that in humans, weight reduction might be a required factor for enhancing cardiovascular and metabolic health. The effectivity of TRE might thus largely depend on study settings, cohort descriptions, and especially timing of the eating windows. However, this has not been investigated in direct comparative studies, and there may be strong individual differences (e.g., with regard to the chronotype).

In summary, while in rodent studies, which are experimentally well controllable, some health‐promoting effects of IF can be uncoupled from CR and weight reduction; the data from human studies are far less clear on this topic. For now, little evidence exists that in humans, intermittent CR harbors superior effects over continuous CR. This conclusion might change with new studies reported rapidly. However, the outcomes of such comparative studies are to be implemented into the body of evidence while taking into account the similarities and differences in duration and specific intervention protocols, adherence rates, as well as the metabolic and nutritional constitution of the cohorts. Ultimately, for everyday life, it might be less important, which dietary intervention elicits superior effects in clinically controlled settings rather than what dietary intervention the individual patient is capable and willing to follow.

## Physiological effects and potential clinical applications of fasting and CR

**Figure 4:**
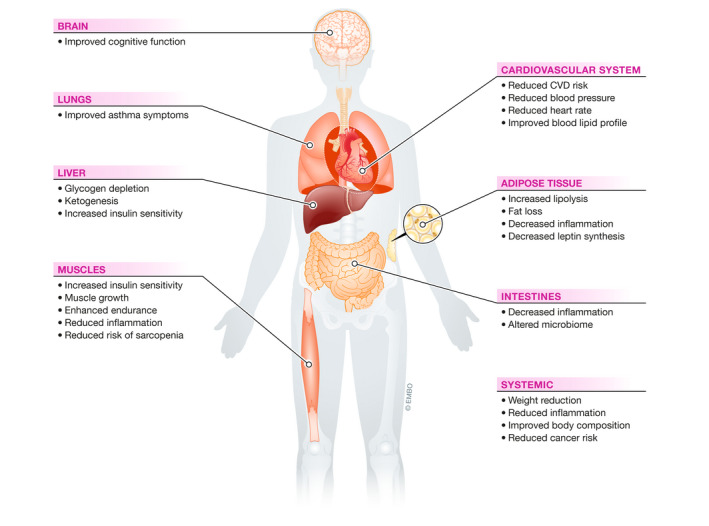
Human organs affected by CR and fasting and selected health benefits Evidence from amounting clinical trials point towards systemic health benefits of CR and IF in humans. A selection of key observations and important metabolic events is presented.

Pre‐clinical and clinical trials suggest a broad range of potential application areas for CR and fasting interventions. However, the individual response to calorie deprivation, the optimal level, and duration for specific clinical settings and other determinants of clinical effectiveness often remain to be clarified. Thus, apart from weight management, specific CR interventions are not yet routinely recommended for specific pathological conditions. Based on pre‐clinical and human studies, we here outline possible future areas of clinical application for CR and IF protocols (Figure 4).

### Obesity and weight reduction

Overweight and obesity are implicated in many complex disease states, including cancer, CVDs, and metabolic diseases, negatively impact quality of life and aggravate other diseases (Lee & Dixon, 2017). Despite large‐scale efforts by public health systems to reduce obesity cases, they are still at all‐time highs, with more than 2 billion people affected worldwide (González‐Muniesa *et al*, 2017). IF and CR are at the forefront of tackling obesity, with safety generally accepted for weight loss management in overweight cohorts (Harvie & Howell, 2017). Thereby fat tissue commonly decreases at a higher rate than lean mass (e.g., ADF in Stekovic *et al*, 2019; CR in Most *et al*, 2018). Typical weight reduction rates of normal weighted to overweight cohorts in clinical trials are −3 to −5% after 4 weeks of ADF and up to −7% after 6 months of CR or ADMF (Johnstone, 2015; Trepanowski *et al*, 2017). The rate of weight loss during complete fasting is estimated at 0.9 kg/day during the first days, which then continuously slows down (Brandhorst & Longo, 2016; Lessan & Ali, 2019). After an initial weight‐loss phase over 6–12 months, bodyweight usually stabilizes during long‐term CR interventions in normal‐ to overweight cohorts under study settings (Das *et al*, 2007; Dorling *et al*, 2020).

### Type 2 diabetes and metabolic syndrome

Continuously elevated insulin and glucose blood levels are hallmarks of type 2 diabetes and pre‐diabetes, and both CR and IF have been shown to reduce insulin levels (Gabel *et al*, 2019). Likewise, markers of systemic inflammation and oxidative stress, two drivers of aging and metabolic diseases, decrease, while immune competency is largely maintained (Meydani *et al*, 2016; Stekovic *et al*, 2019; Wilhelmi de Toledo *et al*, 2020a). However, conflicting data exist on fasting‐induced insulin sensitivity, showing either improvement (ADMF, PF, Furmli *et al*, 2018; eTRF, Sutton *et al*, 2018) or no effect (ADMF, Trepanowski *et al*, 2017) in diverse cohorts. Study design, duration, cohort descriptions, dietary counseling, general diet composition, and other factors might obscure a consistent outcome on insulin parameters. Fasting might also act preventively on diabetes risk (TRE, Wilkinson *et al*, 2020): for instance, in a cohort of overweight older patients (66 ± 10 years), those who periodically fasted had lower prevalence of diabetes (Horne *et al*, 2012). In addition, various fasting regimes show significant visceral fat reduction, which has been linked to increased pre‐diabetes and type 2 diabetes risk (Neeland *et al*, 2012).

However, at this time, IF in diabetic patients is under controversial debate (Barnosky *et al*, 2014; Horne *et al*, 2020). Harsh fasting interventions, for instance, bear the risk of hypoglycemia, a major risk for diabetic patients, which might be less problematic in CR protocols, for which a higher number of trials has been published, showing promising outcomes on insulin parameters (Larson‐Meyer *et al*, 2006; Most *et al*, 2017a).

### Cardiovascular diseases

CR and IF improve cardiac function and cardiovascular parameters in rodents and humans. For instance, blood pressure, CRP, TNF‐alpha and ‐beta levels, and heart rate are generally lower, as exemplified in a study of long‐term CR followers (Meyer *et al*, 2006). Similar observations were made for a long‐term ADF cohort (Stekovic *et al*, 2019) and in shorter interventional studies for nearly all dietary interventions discussed in this review. Both IF and CR improve the blood lipid profile (reduced levels of triglycerides and HDL, increased LDL and VLDL) and other CVD‐relevant factors in non‐obese and obese cohorts (Most *et al*, 2017a, 2018; Trepanowski *et al*, 2017; Francesco *et al*, 2018; de Cabo & Mattson, 2019). Fasting length and baseline characteristics, such as BMI, age, and glucose levels, have an impact on blood pressure effects (long‐term fasting, Grundler *et al*, 2020). Overall, the impact of dietary regimens on the cardiovascular system may lower long‐term CVD risk, at least in healthy individuals, as shown for ADF (Stekovic *et al*, 2019) and CR (Lefevre *et al*, 2009) and mirrored by the low CVD rate in CR societies and the Okinawan people (Willcox & Willcox, 2014).

### Inflammatory diseases and the immune system

Systemic inflammation is central to aging processes, interferes with health in multiple ways, and may be a result of chronic excess calorie consumption (Franceschi *et al*, 2018; Flanagan *et al*, 2020). CR has been shown to reduce inflammatory processes in rodents, primates, and humans (Flanagan *et al*, 2020). IF shows similar outcomes in humans (Patterson & Sears, 2017), although the effects have not been systemically studied. Consistently, small‐scale studies have associated IF with improved symptoms of asthma (Johnson *et al*, 2007), multiple sclerosis, and arthritis (Müller *et al*, 2001; Fitzgerald *et al*, 2018). Extreme CR with malnutrition might elicit negative effects on immune function (Most *et al*, 2017a). Moderate CR without malnutrition as well as long‐term IF with proper dietary compositions reportedly seem safe regarding baseline immune function (CR, Most *et al*, 2017b; ADF, Stekovic *et al*, 2019; TRE, Faris *et al*, 2012; CR, Meydani *et al*, 2016), while acute challenges by infections and susceptibility toward infections may yield different immune responses upon CR and IF, hence medical supervision is advised.

### Cancer

CR delays and diminishes the occurrence of induced and spontaneous cancer throughout aging in mice (Weindruch, 1992; Brandhorst & Longo, 2016) and rhesus monkeys (Colman *et al*, 2009; Mattison *et al*, 2017). Similar observations have been made for IF in rodents. In humans, CR may have a similar impact as suggested by the reduced cancer rates of Okinawan people (Kagawa, 1978; Willcox *et al*, 2007). Extended fasting durations (> 13 h) during nighttime have been associated with reduced breast cancer risk (Marinac *et al*, 2015, 2016). Collectively, CR and IF seem to bear anti‐neoplastic potential, but decisive clinical trials are rare and urgently needed.

Additionally, CR, IF, and short‐term fasting might render tumorous cells vulnerable for chemotherapeutic treatment (Brandhorst & Longo, 2016; de Cabo & Mattson, 2019), as evidenced in cell culture (Safdie *et al*, 2012) and rodent models (Safdie *et al*, 2012; Pietrocola *et al*, 2016) of different cancer types. This selective sensitization may follow a dual impact on cancer cells (e.g., through impaired proliferation due to reduced nutrient availability) and immunogenicity (e.g., through enhanced immunosurveillance via T lymphocytes). In accordance, a small‐scale study in humans reported general safety and tolerability of short‐term fasting (up to 140 h prior to and/or 56 h after chemotherapy) as a complementary treatment to chemotherapy, reducing its side effects while not preventing chemotherapeutic tumor reduction (Safdie *et al*, 2009). Other studies have corroborated these safety findings (reviewed in de Groot *et al*, 2019), warranting further research into the molecular basis and applicability of combining chemotherapy with various forms of fasting (Brandhorst & Longo, 2016; de Groot *et al*, 2019).

### Neurological diseases and cognitive function

The effects of CR and IF on neuronal cells and tissue are well established in animal models, promoting, e.g., increased stress resistance, autophagy induction, DNA repair, and mitochondrial function (Martin *et al*, 2006; Liu *et al*, 2019). In animal models of neurodegenerative diseases, such as Alzheimer’s and Parkinson’s disease, CR and IF can delay the onset and progression of the disease phenotypes (Mattson *et al*, 2017), evoking molecular, physiological, and cognitive improvements (Wang *et al*, 2005; Halagappa *et al*, 2007). 3xTg‐AD mice, a common model of Alzheimer’s disease, maintained for 1 year on either ADF or 40% CR, performed better in behavioral and cognitive tasks, compared to *ad libitum*‐fed animals (Halagappa *et al*, 2007). Moreover, different studies have shown that CR reduced levels of Aβ and Tau accumulation in the brains of disease models (Patel *et al*, 2005). A 6‐month study applying 30% CR to a primate model of Parkinson´s disease found significantly higher levels of locomotor activity in the intervention group, along with reduced dopamine and dopamine metabolites depletion as well as increased survival of dopaminergic neurons in the *substantia nigra* (Maswood *et al*, 2004).

Only few clinical trials have been conducted in this realm: For instance, a 3‐month 30% CR study found significant improvements in an elderly cohort, correlating with reduced insulin and CRP levels (Witte *et al*, 2009). The same group corroborated the findings in a cohort of obese women and also detected increased gray matter volume (Prehn *et al*, 2017), *inter alia* in the hippocampus, an area known to be affected by aging (Bettio *et al*, 2017). The cognitive improvements were found only during the weight loss phase (8 weeks of CR), and not after a 4‐week isocaloric weight maintenance phase, although the weight loss persisted (−10%) (Prehn *et al*, 2017). Consistently, obese patients with mild cognitive impairment showed improvements after a 12‐month‐long CR intervention (Horie *et al*, 2016). The effects of CR on memory function seem to apply to non‐obese cohorts as well, as a multi‐center study found spatial working memory improvements in healthy volunteers after 12 and 24 months of CR (Leclerc *et al*, 2020).

### Ischemic injuries and surgical procedures

Pre‐clinical evidence suggests that IF and CR can protect different tissue types from ischemic injury and surgical damage (Mitchell *et al*, 2010; Jongbloed *et al*, 2014; Rickenbacher *et al*, 2014; Rohrbach *et al*, 2014; Guo *et al*, 2018; Rojas‐Morales *et al*, 2019; Zhang *et al*, 2019). This is supported by first clinical trials. A multi‐center study found that dietary pre‐operative weight loss reduces post‐operative complications in obese patients undertaking bariatric surgery (Van Nieuwenhove *et al*, 2011). Similar effects have been proposed for other surgery types, such as vascular procedures (Mitchell *et al*, 2013).

### Microbiome‐associated diseases

The human microbiome has a decisive role in health and disease, and dysregulated microbiota are associated with CVDs, inflammatory bowel disease, atopic asthma, behavioral disorders, obesity, type 2 diabetes, and autoimmune diseases (Shreiner *et al*, 2015; Durack & Lynch, 2019). The gut microbiome is intrinsically linked to the human diet and metabolome. Accordingly, it is very susceptible to dietary modification, although the exact relationships remain unsatisfactorily understood (Durack & Lynch, 2019).

IF and CR impact the composition and quantity of the microbiome. For instance, CR decreases the ratio of Firmicutes/Bacteroidetes and increases Lactobacilli in the rat microbiome. Some effects are already reversed 1 week after switching back to *ad libitum* feeding, arguing for a highly flexible diet–microbiome axis (Tanca *et al*, 2018). It is believed, that upon CR, the abundance of probiotic strains (e.g., Lactobacillus and Bifidobacterium) increases while that of pro‐inflammatory strains decreases (Zheng *et al*, 2018). As for IF regimens, a trial found decreased abundance of Fusobacterium, a strain associated with colorectal cancer, after 7 days of fasting (He *et al*, 2019). A 10‐day study of very low calorie fasting found reduced abundance of *Lachnospiraceae* and *Ruminococcaceae*, which are known to degrade polysaccharides, and increase levels of Bacteroidetes and Proteobacteria (Mesnage *et al*, 2019). These changes were partly reversed 4 days after ending the study and returned to baseline composition 3 months later.

In turn, differences in the microbiome might affect the effectiveness of CR or IF. For example, higher pre‐intervention abundance of *Akkermansia muciniphila* in obese people was associated with improved CR outcome (glucose homeostasis, insulin sensitivity, body fat distribution, and blood lipids; Dao *et al*, 2016). Interestingly, microbiota‐free mice do not experience the same extent of CR‐mediated effects (e.g., body weight and fat loss) (Wang, *et al*, 2018). Thus, as a determinant of health and disease, the diet–microbiome–host axis emerges as an important factor in shaping CR and fasting‐induced outcomes (Grajeda‐Iglesias *et al*, 2021).

### Exercise and muscle growth/maintenance

In mice, CR has been shown to (counterintuitively) ameliorated age‐induced muscle loss (sarcopenia), although confounding effects, such as increased spontaneous behavior, could also have influenced this observation (van Norren *et al*, 2015). A small‐scale clinical trial found endurance and strength improvements upon TRF (4 h eating windows on 4 days a week) during 8 weeks of workout (3 days a week) when compared to non‐TRF training. Lean mass was retained and no adverse effects on training efficacy were observed (Tinsley *et al*, 2017). However, CR has been reported to have adverse effects on muscle strength in older cohorts (Weiss *et al*, 2007). Hence, more clinical trials are needed to evaluate the physiological effects of combining CR, IF, and exercise on different muscles under varying conditions (Zouhal *et al*, 2020).

### Age and associated diseases

Pre‐clinical data show robust effects of CR and IF on health‐ and lifespan across species (Fontana, 2009; Most *et al*, 2017a; Francesco *et al*, 2018; Madeo *et al*, 2019). In humans, involuntary phases of CR have been reported with two notable examples (WWI in Denmark for 2 years and WWII in Oslo/Norway for 4 years), in which malnutrition was avoided by dietary plans causing a 30% death rate reduction compared to pre‐war times (Hindhede, 1920; Strøm *et al*, 1951). On the Japanese island of Okinawa, inhabitants naturally consumed up to 17% less calories than the rest of Japan, resulting in a markedly increased life expectancy (Willcox *et al*, 2007). Notably, dietary changes due to Westernization abolished this increase, already within the timeframe of one generation. Importantly, most of the age‐associated diseases, some of which have been briefly discussed above, are ameliorated by CR and IF in rodents and humans.

Pre‐clinical studies have shown that the age of onset of IF and CR interventions can influence the effects on health‐ and lifespan. In mice, the lifespan‐extending effects of IF (Goodrick *et al*, 1990) and CR (Duriancik *et al*, 2018; Vaughan *et al*, 2018) are diluted when the regime is introduced later in life. Therefore, future clinical trials and long‐term observational studies, which also consider the age of onset, are necessary to clarify whether any of the discussed dietary interventions lead to increased health‐ and/or lifespan in real‐life, self‐adherent CR and IF populations.

## Considerations and potential caveats of CR and fasting in humans

CR and IF may generally be problematic for minors, very old people, pregnant or breastfeeding mothers, anorectic, or very lean persons, those with bone (density) loss, as well as for special disease groups.

### Hunger, adherence, and mental health during CR and fasting as points to consider

Most people will experience various magnitudes of hunger when cutting calories. A complex regulatory system involving hormones that communicate between the gastrointestinal tract and the brain defines the balance between appetite and satiety. On the one hand, the peptide hormone ghrelin provokes a feeling of hunger via the hypothalamus. Recently, Acyl‐CoA‐binding protein (ACBP) has been proposed to be another phylogenetically conserved appetite‐stimulatory “hunger factor,” which is secreted upon starvation and triggers food intake and obesity (San‐Pedro *et al*, 2019; Charmpilas *et al*, 2020; Madeo *et al*, 2020). On the other hand, adipocyte‐derived leptin counteracts ghrelin’s actions by binding to the LepR receptor in the hypothalamus, provoking satiety. The subjective experience of hunger is often said to decrease with the length of CR and fasting periods, which is in agreement with theories that the ghrelin/leptin system can dynamically adapt to different metabolic and environmental statuses (Davis, 2018). Obesity is linked to dysregulation of this system and resistance to these hormones (Cui *et al*, 2017). The hunger feelings reported in IF and CR studies include a wide range from little/none to considerable/strong (ADF, Heilbronn *et al*, 2005; two‐day fasting, Solianik & Sujeta, 2018; ADMF, Johnson *et al*, 2007) with some studies explicitly noting the absence of hunger feelings in the majority of probands (long‐term fasting, Anton *et al*, 2009; Wilhelmi de Toledo *et al*, 2019). This discrepancy in the literature is likely due to differences in assessing hunger parameters, but also due to different intervention protocols, durations, pre‐intervention dietary behaviors, metabolic states, and motivations, which may affect hunger perception. However, to our knowledge, a systematic analysis of hunger perception and its development over time or its dependency on environmental and nutrition factors for CR or IF is yet missing. Also, it remains unclear whether hunger perceptions may be needed for some of the interventions’ effects.

The adherence rate to different types of CR and IF co‐defines their potential for medical uses. In long‐term CR trials, compliance rates decline over time, and factors such as individual/group counseling, regular monitoring, and diet tracking improve compliance (Flanagan *et al*, 2020). In the CALERIE‐2 study, adherence dropped markedly after 20 weeks of targeted 25% CR (Dorling *et al*, 2020). Reported dropout rates in clinical studies range between single‐digit percentages to 40%. A 1‐year study with obese probands found 38% dropouts in ADMF, 29% in CR, and 26% in the *ad libitum* control group (Trepanowski *et al*, 2017). It is yet unclear, whether intermittent or continuous forms of CR provoke better compliance rates. This likely depends on cohort characteristics and study settings, and may be improved by dietary counseling availability, food provisions, and individual monitoring of study participants.

Severe and malnourished CR has been shown to cause psychological stress, depression, and a detrimental impact on mental and sexual health. Instead, controlled CR or fasting with adequate nutritional compositions is not generally reported to have negative outcomes on quality of life, mood, or other psychological parameters (Velthuis‐te Wierik *et al*, 1994; Martin *et al*, 2016; Wilhelmi de Toledo *et al*, 2019). A study of long‐term fasting reported a gradual rise in emotional and physical well‐being (Wilhelmi de Toledo *et al*, 2019). However, the discriminators among positive, neutral, and negative psychological outcomes are highly individual and subject to the intervention’s magnitude and length. Thus, psychological monitoring should be sought through extended phases of CR or fasting, and should be mandatory for individuals who are endangered or susceptible to depression or other mental diseases.

### Lean mass loss, weight regain, and potential bone density issues during and after CR and IF

Loss of lean mass usually occurs during CR and IF, but at a slower rate than fat tissue (Trepanowski *et al*, 2017). Observations of a long‐term cohort of ADF followers did not reveal differences in body composition to a weight‐matched, fasting‐naive control group (Stekovic *et al*, 2019). The CALERIE studies showed reductions in lean mass in both young and older participants, while a negative impact on muscle strength was only observed in older groups performing CR (Weiss *et al*, 2007; Larson‐Meyer *et al*, 2010). Controversially, a recent late‐TRE study found no significant decrease in fat mass, but in lean mass (Lowe *et al*, 2020). Overall, further research in this area is needed, especially in elderly cohorts at risk for age‐associated sarcopenia (Walston, 2012). Complementary exercise is believed to halt some of the lean mass loss that occurs during long‐term CR or IF and may promote fat reduction (Keenan *et al*, 2020), which may be a viable option in clinically subscribed dietary interventions.

Follow‐up data on weight regain are rare with mixed outcomes. Upon re‐assessing energy demand after 6 months of CR or ADF, modest weight regain was reported in both groups (Trepanowski *et al*, 2017). Importantly, during the CALERIE trial, the participants did not develop eating disorders or tendencies toward binge eating (Williamson *et al*, 2008), which could potentially annulate long‐term health benefits and weight loss in overweight patients.

Data on changes in bone mass and/or density due to sustained CR seem contradictory. In non‐obese, healthy participants, ADF was reported to have no effect on bone mass or density in a 4‐week RCT or in a cohort of self‐adherent ADF followers, who performed the intervention for several months (Stekovic *et al*, 2019). Likewise, 6‐month CR did not decrease bone mass in the CALERIE‐1 study (Redman *et al*, 2008), whereas CALERIE‐2, lasting for 2 years, did find a significant decrease in some areas (Villareal *et al*, 2016). The intervention magnitude and length, nutritional composition, complementary exercise, and the age of the participants likely have a big impact on bone health parameters. Future long‐term studies on IF and CR need to address the effects on bone health in more detail, especially in elderly cohorts, and the possible use of nutrient supplements to support bone structure.

### The immune system during CR and IF

In mice, chronic CR increases the susceptibility to infections, especially of viral nature (Gardner, 2005; Kristan, 2008). In humans, the basic cellular immune profile does not differ significantly between long‐term ADF and control groups (Stekovic *et al*, 2019), while inflammatory markers may even be reduced. One study found slightly reduced immune cell counts after Ramadan fasting (Faris *et al*, 2012), while another 6‐month CR study found improved T‐cell function (Ahmed *et al*, 2009). Hence, future studies need to address the effects of CR and IF on the immune system in real‐life settings, especially in elderly populations and under acute pathogenic challenge.

### CR with malnutrition and starvation

Correctly applied CR and IF, per definition, exclude malnutrition. In clinical trials, this can be prevented by dietary counseling, pre‐defined meals, and/or monitoring of dietary behavior. In non‐trial private endeavors, dietitians should be consulted before undertaking interventions. Due to ethical considerations, malnutrition and starvation clinical studies remain questionable. Insights have been gained from observational studies: In the “Minnesota Starvation Experiment,” extreme CR (40%) with improper dietary composition (e.g., insufficient protein, vegetable and fruit intake) and increased physical activity in young men over 6 months resulted in approx. 25% weight loss (2/3 fat, 1/5 fat‐free mass), chronic fatigue, lower limb edema, severe emotional distress, depression, confusion, apathy, suicidal thoughts, and libido loss. Most of the effects were normalized upon refeeding (KEYS *et al*, 1950). Furthermore, malnourished, extreme CR may negatively impact reproductivity and fecundity, steroid production, ovarian function, and immune function (Most *et al*, 2017b) (Ulijaszek, 1996).

Several early studies report adverse events in long‐term fasting of obese patients, although it was noted that many of those are absent upon minimal supplementation with carbohydrate and/or protein (*Br Med J*, 1978). These adversities include decreased bone density, acidosis, liver dysfunction, menses dysregulation, baldness, edema, nausea, headaches, fatigue, depression, kidney failure, cardiac problems, and even death, among others (Harrison & Harden, 1966; Hermann & Iversen, 1968; Ross *et al*, 1969; Rooth & Carlström, 1970; Munro & Duncan, 1972; *Br Med J*, 1978; Devathasan & Koh, 1982). A recent analysis found few adverse events for prolonged water‐only fasting (median 7 days, range 2–28 days) and concluded that it is, in principle, safe for multiple purposes (Finnell *et al*, 2018). Modern (short‐term) IF or CR studies under well‐nourished conditions that sufficiently provide micro‐ and macronutrients, usually show no strong adverse effects.

## Alternative approaches to CR and fasting

Fasting and CR may be unsuitable in certain settings and, as mentioned above, for specific groups, including people of advanced age, with multiple co‐morbidities or minors. Furthermore, real‐life adherence of eligible participants to these interventions might be lower without trial‐associated counseling and motivational support. Thus, increasing efforts aim at developing alternative approaches that elicit (some) fasting‐ or CR‐like effects without the need to reduce calorie intake. One of them is bariatric surgery, which aims at weight loss and comorbidities management (Benaiges *et al*, 2015; Wolfe *et al*, 2016). Another, less invasive approach is the concept of pharmacological supplementation with CRMs, which have been extensively reviewed elsewhere (Ingram *et al*, 2006; Lee & Min, 2013; Mariño *et al*, 2014; Madeo *et al*, 2019; Hofer *et al*, 2021; Ingram & Roth, 2021).

Specific types of dietary modulation also represent alternative avenues for persons who cannot adhere to CR or IF protocols. These include, among others, the fasting‐mimicking diet (FMD; Brandhorst *et al*, 2015; Choi *et al*, 2016b), ketogenic diets (Sampaio, 2016; Boison, 2017; Ludwig, 2020), as well as very low calorie (Kim, 2021), low‐carbohydrate, or low‐fat diets (Kim, 2021).

The FMD, for instance, is commercially available as a low‐calorie, ‐sugar, and ‐protein diet that contains high levels of unsaturated fats and micronutrients (Brandhorst *et al*, 2015; Choi *et al*, 2016a; Wei *et al*, 2017). In both pre‐clinical and clinical studies, it has shown promising effects on health parameters, including overall body weight, body fat, trunk fat, IGF‐1, blood pressure, blood lipids, and CRP. Thereby, higher risk subgroups (by means of weight, blood pressure, or blood lipid profile) generally benefited more than lower‐risk groups (Wei *et al*, 2017). In pre‐clinical studies, it was effective in ameliorating age‐induced weight gain, improving cognitive function and locomotion, elevating adult neurogenesis, and increasing median lifespan of mice (Brandhorst *et al*, 2015). In agreement with its low‐calorie basis, the FMD has been shown to significantly increase blood KB levels (Brandhorst *et al*, 2015).

Initially developed as a treatment option for epilepsy, ketogenic diets share similarities with the FMD and other low‐carbohydrate diets. To shift the metabolism to a ketogenic state, they pair a drastic reduction in carbohydrates with an increase in fat (> 70%), forcing the body to produce KBs. Ketogenic diets are being studied for a range of maladies, including cancer, CVDs, diabetes, obesity, neurological diseases, multiple sclerosis, and others (Bahr *et al*, 2020; Ludwig, 2020; Ferrere *et al*, 2021). They are generally believed to be safe (Ludwig, 2020) if done in an intermitted fashion and if formulated well, and hold promise for clinical translation. Similar to IF and CR, ketogenic diets can induce autophagy and extend health‐ and lifespan in model organisms (Roberts *et al*, 2017; Wang, *et al*, 2018). Many of these effects may eventually originate from the increase in KBs, which *per se* have autophagy‐inducing and lifespan‐prolonging effects (Edwards *et al*, 2014; Camberos‐Luna *et al*, 2016; Veech *et al*, 2017; Han *et al*, 2020; Torres‐Esquivel *et al*, 2020).

While all herein discussed alternative approaches show promising results, future research will establish their long‐term effects and possible pitfalls, which will ultimately decide their clinical feasibility. It will be of great interest to see whether combining IF or CR with CRMs might induce synergistic metabolic effects. Variations in timing of the pharmacological/dietary supplementation might yield interesting new insights, e.g., giving a substance during fasting or *ad libitum* eating to constantly and timely modulate various molecular pathways (autophagy, ketone metabolism, mTOR activity, etc.). If supplemented during eating periods, CRMs could constantly shift the metabolism toward a fasting‐like state, minimizing the effort to cut calories. If taken during fasting periods, non‐caloric supplements that *per se* induce autophagy or ketogenesis might synergistically elevate a fasting response. A further prospect is to develop individual programs for patients based on their (health) goals and personal genetic or microbial setting, which will likely be more manageable by combining knowledge from all aforementioned alternatives.

## Conclusion and emerging questions

Over the recent years, IF and CR have gained scientific, medical, and public attention due to their potentially broad health benefits. In preclinical studies, fasting and CR have been shown to prolong life‐ and healthspan, induce autophagy, and ameliorate symptoms of various diseases, such as CVDs, type 2 diabetes, neurodegenerative diseases, cancer, or ischemic injuries. However, transition into the clinics has been slow. Importantly, most clinical trials in the field last for a few weeks to several months, but long‐term and follow‐up trials are required to evaluate the effects and safety in the long run. Furthermore, the great majority of IF and CR trials have focused on overweight, metabolically compromised and/or middle‐aged cohorts. Thus, a generalization of the findings to the wider population remains difficult. Still, as summarized herein, the current understanding is that the downsides are relatively small for most people and the potential benefits may well outweigh the disadvantages.

Important questions remain partly unanswered: Are there molecular and physiological benefits of one intervention type over another when calories are matched, apart from individual preferences? For IF, which are the molecular improvements beyond mere weight loss? How do circadian rhythm and eating/fasting time patterns interact? Can we maximize the effectiveness by personalizing fasting or CR? Which baseline characteristics influence the outcome/response to fasting and CR? Can we support medical treatments (e.g., chemotherapy, surgeries) by applying fasting or CR? How do recurring fasting or CR phases affect the likelihood to develop metabolic or age‐associated diseases?

Eventually, we might see health care systems incorporating IF‐ and CR‐like therapies into routine care. First steps toward this development are emerging with scientific suggestions for therapeutical application (de Cabo & Mattson, 2019), mirrored by the rising popularity of wellness and/or rehabilitation centers that provide medical counseling during special fasting applications (Wilhelmi de Toledo *et al*, 2019). On a different line, deepening our understanding of the molecular basis of fasting and CR will help discover and develop novel CRMs (Madeo *et al*, 2019) that might overcome some of the application obstacles in humans. Since many fasting‐ and CR‐affected pathways also play major roles in the process of aging, research in these areas is extending our understanding of healthy aging and the underlying physiological and molecular foundations.

## Author contributions

SJH and FM conceptualized the review. SJH wrote the manuscript, and SJH, DC‐G and MIM designed the figures. SJH, DC‐G, MIM and FM contributed to the editing and proofreading of the final draft.

## Conflict of interest

F.M. and D.C.‐G. are scientific cofounders of Samsara Therapeutics, a company that develops novel pharmacological autophagy inducers. F.M. has equity interests in and is advisor of TLL, The Longevity Labs GmbH. D.C.‐G. has equity interests in TLL, The Longevity Labs GmbH. S.J.H and M.I.M. declare that they have no conflict of interest.

**Pending issues**

1. Determination of molecular and physiological differences between CR and different forms of IF in humans.
2. Do controlled IF and CR interventions change emerging indirect markers of aging, e.g., epigenetic clocks, in humans?
3. Long‐term clinical trials and follow‐up studies to establish the effects of CR and IF on the development and progression of metabolic‐ or age‐associated diseases in different human cohorts.
4. More clinical trials concerning neurodegenerative disorders (e.g., in early‐onset AD patients), CVDs, and other age‐associated diseases.
5. More research on the real‐life applicability of CR and IF as supportive measures before or after medical treatments, such as surgeries or chemotherapies.
6. Understanding the impact of circadian rhythm on eating/fasting patterns.
7. Which molecular and physiological effects observed in IF studies can be uncoupled from weight loss/CR in humans?
